# Anti-Claudin Treatments in Gastroesophageal Adenocarcinoma: Mainstream and Upcoming Strategies

**DOI:** 10.3390/jcm12082973

**Published:** 2023-04-19

**Authors:** Giulia Grizzi, Kostantinos Venetis, Nerina Denaro, Maria Bonomi, Andrea Celotti, Antonia Pagkali, Jens Claus Hahne, Gianluca Tomasello, Fausto Petrelli, Nicola Fusco, Michele Ghidini

**Affiliations:** 1 Operative Unit of Oncology, ASST of Cremona, 26100 Cremona, Italy; 2 Division of Pathology, IEO, European Institute of Oncology IRCCS, 20141 Milan, Italy; 3 Department of Oncology and Hemato-Oncology, University of Milan, 20122 Milan, Italy; 4 Oncology Unit, Fondazione IRCCS Ca’ Granda Ospedale Maggiore Policlinico, 20122 Milan, Italy; 5 Department of Surgery, ASST of Cremona, 26100 Cremona, Italy; 6 School of Medicine, National and Kapodistrian University of Athens, 11527 Athens, Greece; 7 Division of Molecular Pathology, The Institute of Cancer Research, London SM2 5NG, UK; 8 Oncology Unit, Medical Sciences Department, ASST Bergamo Ovest, 24047 Bergamo, Italy

**Keywords:** gastric cancer, claudins, anti-claudin 18.2, esophageal cancer, zolbetuximab, car-T cells, chemotherapy

## Abstract

Claudins (CLDNs) are a multigene family of proteins and the principal components of tight junctions (TJs), which normally mediate cell–cell adhesion and selectively allow the paracellular flux of ions and small molecules between cells. Downregulation of claudin proteins increases the paracellular permeability of nutrients and growth stimuli to malignant cells, which aids the epithelial transition. Claudin 18.2 (CLDN18.2) was identified as a promising target for the treatment of advanced gastroesophageal adenocarcinoma (GEAC), with high levels found in almost 30% of metastatic cases. CLDN18.2 aberrations, enriched in the genomically stable subgroup of GEAC and the diffuse histological subtype, are ideal candidates for monoclonal antibodies and CAR-T cells. Zolbetuximab, a highly specific anti-CLDN18.2 monoclonal antibody, demonstrated efficacy in phase II studies and, more recently, in the phase III SPOTLIGHT trial, with improvements in both PFS and OS with respect to standard chemotherapy. Anti-CLDN18.2 chimeric antigen receptor (CAR)-T cells showed a safety profile with a prevalence of hematologic toxicity in early phase clinical trials. The aim of this review is to present new findings in the treatment of CLDN18.2-positive GEAC, with a particular focus on the monoclonal antibody zolbetuximab and on the use of engineered anti-CLDN18.2 CAR-T cells.

## 1. Introduction

Gastroesophageal cancer (GEAC) is a highly aggressive malignancy that can be resistant to treatment, with a poor prognosis and few effective targeted therapies. Classification of GEAC includes anatomic location, histology and molecular features according to The Cancer Genome Atlas (TCGA) classification [1]. At present, the role of molecular pathology is challenging, because more information is needed to tailor an adequate treatment, such as human epidermal growth factor receptor 2 (HER2) status, combined positive score (CPS)–programmed death ligand 1 (PDL1) expression, and MSI or mismatch repair (MMR) deficiency.

Treatment involves a multidisciplinary team, taking into consideration histology, molecular characteristics, tumor stage, patient’s conditions, and the will and expertise of the center [2]. In stage II/III, perioperative chemotherapy plus surgery or a surgical approach followed by adjuvant treatment are preferred. In unresectable GEAC, the first-line therapy includes chemotherapy plus immunotherapy in HER2-negative CPS-PDL1-positive (≥5) tumors [3] or a combination plus trastuzumab in HER2-positive disease [4]. In chemorefractory HER2-positive cancer, randomized trials have demonstrated the efficacy of the antibody–drug conjugate trastuzumab deruxtecan (T-DXd) [5]. Additionally, in pretreated patients, new perspectives include the combination of immunotherapy and anti-angiogenetic drugs, such as lenvatinib plus pembrolizumab in the phase 2 LEAP-005 trial (NCT03797326) [6]. Despite recent improvements in medical approaches, new molecular targets are urgently needed [7]. The tight junction protein claudin 18.2 (CLDN18.2) was identified as a promising target for the treatment of advanced GEAC. In general, claudin proteins play a fundamental role in the scaffolding for cell–cell adhesion and migration. However, malignant transformation leads to disruption of tight junctions, which exposes CLDN18.2 protein on the surface of tumor cells [8]. The TCGA genomically stable (GS) subgroup is rich in mutations with the fusion of CLDN18–ARHGAP, in addition to the diffuse histological subtype [1]. High levels of CLDN18.2 are found in almost 30% of metastatic GEAC, with a higher prevalence in Asian populations (up to 50% of metastatic GEAC), those of female sex, patients younger than 65 years old, tumors localized in the gastric body, and HER2- and EBV-negative cancers [9,10,11].

The aim of this review is to present new findings for the treatment of CLDN18.2-positive GE cancer, with particular interest in the monoclonal antibody zolbetuximab and in the use of engineered anti-CLDN18.2 chimeric antigen receptor (CAR)-T cells.

## 2. Claudin Proteins: Structure and Molecular Pathway

**Figure 1:**
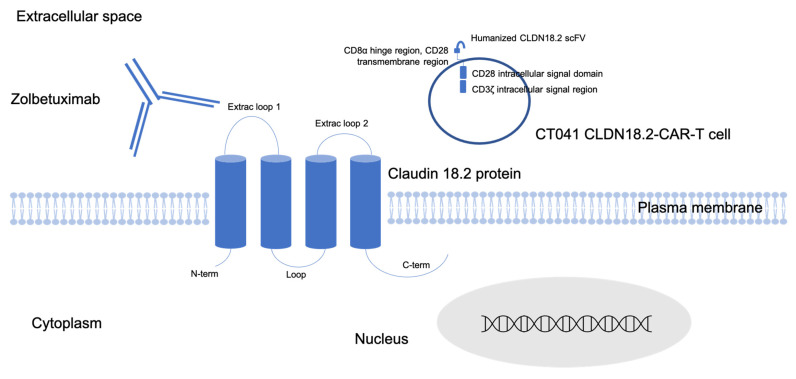
Schematic structure of the claudin18.2 protein located in the tight junction of gastric cells. Claudin18.2 is the target of the monoclonal antibody zolbetuximab and of the CT041 anti-CLDN18.2 CAR-T cell. Extracellular loops: site of interaction for potential therapeutic antibodies. C-term is the site of phosphorylation and interactions with signaling molecules. Legend: C-term: C-terminal domain; Extrac: extracellular; N-term: N-terminal domain; ScFv: single-chain fragment variable.

Claudins (CLDNs) are a multigene family that encode tetraspan proteins and represent principal components of tight junctions (TJs), which normally mediate cell–cell adhesion and selectively allow the paracellular flux of ions and small molecules between cells [12]. CLDNs consist of four transmembrane domains, including a N-terminus and a C-terminus in the cytoplasm and two extracellular loops that span the transmembrane domains [13] (Figure 1).

The claudin family is composed of 24 members, and according to sequence analyses, these proteins are clustered into two separate groups, known as classic CLDNs (1–10, 14, 15, 17, 19) and non-classic CLDNs (11–13, 16, 18, 20–24), based on their degree of sequence similarity [14]. CLDNs are found in normal tissues, including gastric, pancreatic, and lung tissues, following strictly tissue-specific expression patterns [15]. The downregulation of CLDNs increases the paracellular permeability of nutrients and growth stimuli to malignant cells, which aids the epithelial transition [16]. On the contrary, upregulation of certain CLDNs has been found to increase permeability to paracellular markers [17]. Such modifications of the function of CLDNs have been associated with carcinogenesis in the respective tissue [18]. It has been suggested that the loss of claudins and other tight junction proteins in cancer is a mechanism underlying the loss of cell adhesion and a crucial step in the progression of the disease toward metastasis [19].

Among the different CLDNs that have been described in the literature so far, CLDN1–5, CLDN7–12, CLDN16, and CLDN18 are expressed in normal gastric mucosa [20]. Specifically, isoform 2 of CLDN18.2, which is confined to differentiated and stem gastric epithelial cells, is able to regulate paracellular permeability to Na+ and H+ ions [21]. Although in normal tissues, the epitopes of CLDN18.2 within the TJ complex are quite inaccessible, in tumor tissues, these epitopes can be targeted by monoclonal antibodies (mAb), given the disturbed cell polarity that occurs during carcinogenesis [8]. A recent study, which assessed the expression of CLDN18.2 in 481 patients with GEAC, showed that the expression of this protein was associated with certain clinicopathological features, including mucin phenotype, and integrin αvβ5 and lysozyme levels [22]. Moreover, EBV infection in epithelial cells is mediated by cell-to-cell contact, and the presence of extensive cell junctions prevents viral clearance by antibodies. Therefore, the magnitude of claudin expression in GEAC is often associated with EBV infection [21]. By assessing the clinical implications of CLDN18.2 in GEAC patients, Baek et al. revealed a higher expression of this protein in diffuse-type and HER2-positive GEAC, while no association was found between CLDN18.2 expression and survival rates [23]. Evidence provided by the FAST and MONO trials [24,25] on the efficacy of zolbetuximab, a monoclonal antibody against CLDN18.2, in patients with GEAC, makes this protein a unique biomarker for the development of additional therapies [26].

Apart from CLDN18.2, other proteins of this family have been reported as emerging prognostic and predictive biomarkers of GEAC. Among the most relevant, CLDN1 may provide prognostic information, given that its low expression has been associated with a poor prognosis in stage II colon cancer [27]. Low expression of CLDN3 was found to be correlated with positive lymphatic invasion, advanced tumor depth, and lower TNM stage [28], whereas overexpressed CLDN3 was related to lymph node metastasis [29]. Interestingly, high CLDN3 expression was seen in immunologically responsive tumors and negatively correlated with GC CD8+ T cells [30]. A meta-analysis demonstrated that CLDN4 expression was associated with an increased pathological tumor (pT) classification, tumor size, and lymph node metastasis [31]. On the other hand, in patients with GC, abnormal expression of CLDN6 and CLDN10 has been associated with decreased OS and immune infiltration, respectively [32,33]. All these findings confirm the potential prognostic and predictive role of the CLDN protein family, suggesting that further investigations are warranted, in order to fully unravel the clinical value of these markers.

## 3. Anti-Claudin 18.2 Treatments

### 3.1. Anti-Claudin 18.2 Inhibitors

Zolbetuximab (IMAB362) is a chimeric IgG1 monoclonal antibody that binds to CLDN 18.2 on the cell surface and which subsequently induces cancer death through the processes of antibody-dependent cellular cytotoxicity and complement-dependent cytotoxicity. The phase II multicentric MONO study evaluated zolbetuximab monotherapy in the second- and further-line treatment of patients with GEAC. Included patients had moderate-to-strong CLDN 18.2 expression in ≥50% or more of the total tumor cells. Patients were enrolled in two sequential cohorts (cohort 1300 mg/m^2^; cohort 2600 mg/m^2^) and a dose-expansion cohort (cohort 3600 mg/m^2^). The primary endpoint was the objective response rate (ORR), while secondary endpoints were clinical benefit (ORR + stable disease), progression-free survival (PFS), safety, tolerability, and pharmacokinetic profile. Treatment was well tolerated and achieved a 9% overall response rate (ORR) in a population of the 43 patients for whom antitumor activity data were available. In patients with high claudin expression (≥70%), the ORR was 14% [25]. The subsequent phase II randomized FAST trial evaluated zolbetuximab (loading dose, 800 mg/m2 then 600 mg/m^2^ every three weeks) + EOX (epirubicin, oxaliplatin, capecitabine) chemotherapy versus EOX alone in the first-line treatment of advanced GEAC patients with moderate-to-strong CLDN18.2 expression in ≥40% of tumor cells. The primary endpoint was PFS, and the secondary endpoint was OS. A total of 252 patients were randomized into treatments, with both progression-free survival (PFS) (median 7.5 versus (vs.) 5.3 months, hazard ratio (HR) = 0.44; 95% confidence interval (CI), 0.29–0.67; *p* < 0.0005) and overall survival (OS) (median 13 vs. 8.3 months, HR = 0.55; 95% CI, 0.39–0.77; *p* < 0.0005) significantly improved in the combination arm (zolbetuximab + EOX) compared to the EOX arm. The PFS and OS benefits were maintained in patients with a high claudin expression of ≥70%, while no OS advantage was seen in the population with CLDN18.2 expression where 40%–69% of tumor cells were positive. The ORR was 39% in the experimental arm vs. 25% in the control arm (*p* = 0.034) [24]. Therefore, the survival advantage seen in this study was primarily driven by the population with a CLDN 18.2 expression of ≥70% of tumor cells. Interestingly, the control arm of this study (EOX chemotherapy) performed poorly in comparison with previous studies (median survival of 8.3 months in the FAST versus 11.2 months and 11.3 months in the REAL-2 and REAL-3 studies, respectively). This might be explained by CLDN18.2 aberrations being most frequently associated with a genetically stable subgroup of cancers and diffuse histology, with a poorer prognosis. Moreover, the lack of further treatments after the first-line treatment (60% of patients) could be an additional explanation for the poorer outcome of the EOX comparator arm with respect to a previous trial [34]. On the whole, although CLDN18.2 expression retains a predictive role for response to therapy, its prognostic impact is not clear yet [24]. Indeed, while study results suggested that a reduction of expression of CLDN 18 may be an independent indicator of poor prognosis in patients with GC [35], the same was not true for esophageal cancer [36].

The ongoing multicohort ILUSTRO phase II trial aims to evaluate the antitumor activity and safety/tolerability of zolbetuximab as a monotherapy, in combination with modified FOLFOX6 (with or without nivolumab) and in combination with pembrolizumab in an unblinded fashion. Cohort 2 in this trial includes previously untreated GEAC patients with high CLDN18.2 expression (≥75% of tumor cells demonstrating moderate-to-strong membranous staining with central IHC testing) and receiving zolbetuximab + mFOLFOX6. With 21 patients enrolled and 19 evaluable for the primary endpoint of response, the ORR was 63.2 (95% CI: 38.4–83.7), the median PFS was 13.7 months (95% CI: 7.4—not estimable), and the 12-month PFS rate was 58%. Two multicenter phase III trials, the SPOTLIGHT and GLOW trials, have concluded enrollment [37]. This phase III double-blind randomized SPOTLIGHT trial compared zolbetuximab plus mFOLFOX6 chemotherapy (mFOLFOX6) with a placebo plus mFOLFOX6 in unresectable or metastatic CLDN18.2-positive (moderate-to-strong membrane staining in ≥75% tumor cells by IHC) and HER-2 negative GEAC. Patients were randomized 1:1 to zolbetuximab 800 mg/m^2^ cycle 1 day 1, followed by 600 mg/m2 cycle 1 day 22 and every 3 weeks in later cycles + mFOLFOX6 (days 1, 15, 29) for four 42-day cycles vs. placebo + mFOLFOX6; patients without progression (PD) continued for >4 cycles with zolbetuximab or placebo, + folinic acid and 5 FU at investigator’s discretion, until PD or discontinuation criteria were met. The primary endpoint was PFS, and secondary endpoints included OS, ORR, and safety. Among the 565 randomized patients, the mFOLFOX6 + zolbetuximab arm showed a significantly longer PFS (median 10.6 vs. 8.7 months, HR 0.75, 95% CI 0.589–0.942, *p* = 0.0066) and OS (median 18.2 vs. 15.5 months, HR 0.75, 95% CI 0.601–0.936, *p* = 0.0053) compared to the control arm, while the ORR was similar between the treatment arms (60.7 vs. 62.1%). Subgroup analyses showed that patients treated with zolbetuximab + mFOLFOX6 had significantly longer PFS and OS for both the diffuse and intestinal Lauren subtypes with respect to the control arm. Differently, patients with mixed and other subtypes gained no advantage from the experimental treatment, in terms of survival outcomes [38]. Conversely, the phase III randomized GLOW trial enrolled 500 patients globally using capecitabine and oxaliplatin (CAPOX) as the chemotherapy partner and comparator. Positivity to CLDN18.2 was assessed, similarly to the SPOTLIGHT trial [39]. Recently, Astellas Pharma Inc. announced positive results for the trial, with statistically significant improvements in both median PFS and OS produced by zolbetuximab + CAPOX. Detailed results will be presented at a future scientific congress and submitted for publication.

On the whole, treatment with zolbetuximab has shown a good tolerability profile in association with chemotherapy. In the phase II MONO and FAST trials, use of zolbetuximab was associated with increased nausea and vomiting with respect to the standard therapy [24,25,40]. In particular, any grade nausea was reported in 61–82% of cases, while vomiting was reported in 50–67.5% of cases [24,25]. Other common reported toxicities were anemia, neutropenia, weight loss, and fatigue [24,25]. In the phase III SPOTLIGHT trial, the most common adverse events reported with zolbetuximab + mFOLFOX6 were nausea (82%), vomiting (67%), and decreased appetite (47%), while the incidence of serious adverse events was similar between both arms (45% vs. 43%) [38]. Table 1 summarizes the available phase II and III trials with the anti-CLDN18.2 monoclonal antibody zolbetuximab in GEAC.

### 3.2. CLDN18.2 CAR T-Cell Therapy

One of the most promising treatments in the immunotherapy landscape is the use of chimeric antigen receptor (CAR)-T cells [41]. This innovative technology allows autologous T-lymphocytes to be isolated from the patient’s bloodstream and modified with the use of viral vectors that enable the introduction of the CAR. This process gives T-lymphocytes the ability to recognize specific tumor-associated antigens. Moreover, costimulatory molecules, such as CD28 and CD137, can be incorporated to increase the functional activation and in vivo survival.

CT041 is a molecule composed of engineered autologous T cells that express the CLDN18.2-specific CAR. CT041 was the first CLDN18.2-CAR-T cell to demonstrate promising preclinical tumor regression in mice bearing a GEAC cell line and in patient-derived tumor xenograft (PDX) models [42]. The first in-human phase I pilot study demonstrated the safety profile of CT041 in seven advanced pretreated GEAC patients [43]. Recently, the interim results of a phase I clinical trial confirmed the acceptable safety profile of CT041 in 37 patients with heavily pretreated gastrointestinal cancers [44]. The most common adverse event was the expected transient hematologic toxicity (grade 3 or higher in all patients), which was mainly related to the preconditioning regimen with fludarabine, cyclophosphamide, and nab-paclitaxel. Grade 1 or 2 cytokine release syndrome (CRS) was observed in 94.6% of patients. In this study there was no evidence of grade 3 or higher, dose-limiting toxicities, or treatment-related deaths. ORR, DCR, and OS outcomes were reported in an exploratory manner in the overall population and in GC patients. In the 28/37 patients affected by GEAC, the ORR and disease control rate (DCR) reached 57.1% and 75.0%, respectively, and the 6-month OS rate was 81.2%. Among these patients, 42.9% presented signet-ring cell carcinoma, 57.1% Lauren diffused/mixed type and 67.9% peritoneal metastasis on baseline CT imaging, which are well-known poor prognostic characteristics.

Recently, safety and efficacy data were presented regarding 14 patients with GEAC and treated with CT041 in a phase Ib/II trial [45]. In the phase Ib part of the study, no dose-limiting toxicities and treatment-related deaths were observed. Hematologic toxicity was the most common grade 3 or higher adverse event, while CRS were mostly of grade 1 or 2, and only one patient experienced a grade 4 injury and then fully recovered. The exploratory efficacy outcomes showed 57.1% partial responses (PR, 8/14 patients), a mPFS of 5.6 months (95%CI 1.9–7.4), and a mOS of 10.8 months (95% CI 5.1–not estimable [NE]). The same safety profile was confirmed in a phase Ib clinical trial in a western population of 11 patients with gastric and pancreatic adenocarcinomas [46]. Despite the obvious limitations related to the small number of patients included, in those who reached the time for assessment, the ORR was 100% (3/3) in the gastric subgroup, including one complete response.

Despite these promising reports, the use of CAR-T cell therapy has some limits. First of all, compared to standard chemo-immunotherapy, it requires a long production process and is more expensive. Second, the effectiveness of CAR-T cell treatment can be compromised by the heterogeneity of tumor antigens and by problems due to the proliferation and stability of the T-cells inside the tumor [47,48]. Furthermore, a hostile tumor microenvironment (TME) with overexpression of inhibitory receptors and the presence of immunosuppressive cells (tumor-associated macrophages, myeloid-derived suppressor cells, and T-regulatory cells) can lead to tumor immune escape.

Currently, many preclinical and clinical trials with CLDN18.2 CAR-T cells are ongoing in CLDN18.2-positive GEAC. A preclinical study using KD-496, a tandem CAR molecule targeting the two tumor-associated antigens NKG2DL and CLDN18.2 was conducted in mice [49]. KD-496 CAR-T cells showed a strong response to gastric cancer in PDX models, with no obvious safety issues. Several early phase clinical trials with specific CLDN18.2 CAR-T cells (NCT03874897, NCT04404595, NCT04966143, and NCT04467853) are ongoing, and these results are expected to significantly modify clinical practice and improve GEAC patient outcomes.

## 4. Conclusions

GEAC remains the second highest cause of cancer-related mortality worldwide, and cytotoxic chemotherapy remains the milestone treatment in a first-line setting for advanced disease. However, during recent years, the therapeutic scenario has been modified by the introduction of specific biomarker-targeted agents in HER2- and PDL1-positive GEAC. However, to date, targeted therapy combined with chemotherapy has prolonged survival in a limited number of patients. In fact, only 15–20% of GEAC patients may benefit from trastuzumab, and only patients with PDL-1 CPS ≥ 5 benefit from nivolumab. However, the increase in treatment costs with the addition of zolbetuximab is counterbalanced by a higher number of treatable patients (roughly 30% of metastatic GEAC).

CLDN18.2 aberrations, which are enriched in the genomically stable subgroup and diffuse histological subtype of GEAC, are ideal candidates for targeted drugs such as monoclonal antibodies and CAR-T cells. Zolbetuximab, a monoclonal antibody that is highly specific for CLDN18.2, demonstrated its efficacy in phase II studies and, more recently, in the phase III SPOTLIGHT trial [38], with an improvement of both PFS and OS with respect to the standard treatment. Anti-CLDN18.2 CAR-T cells showed a safety profile in early phase clinical trials with a prevalence of hematologic toxicity, mainly related to the preconditioning regimen [45]. Despite the limited number of patients enrolled, promising outcome effects were reported in GEAC patients, which need to be confirmed in later-phase trials.

Nevertheless, there are some aspects that we should underline. First, CLDN18.2 expression varies among different ethnicities, with a higher expression in Asian patients (up to 50% in Japanese GEAC) and a lower expression in Caucasian patients (17–20%). Moreover, CLDN18.2′s expression can show intratumor heterogeneity, with different values of positivity for primary disease, nodal, and distant metastases [11]. Moreover, recent reports of the survival benefit with upfront anti-PD1 immunotherapies plus chemotherapy make it difficult for the clinician to choose the right first-line therapy, especially in PDL1- and CLDN18.2-positive disease. Third, CAR-T cell treatment is not immediately feasible in routine clinical practice, because of its cost and the long production process.

In conclusion, the therapeutic landscape of advanced GEAC is changing, in order to offer a tailored strategy to each patient. These recent evidences allow a better chance of survival in CLDN18.2-positive GEAC patients, providing them new treatment options and increased hope of being cured.

## Figures and Tables

**Table 1:** Phase II and III clinical trials with the anti-CLDN18.2 monoclonal antibody zolbetuximab in GEAC.

Study Name	NCT Number	Phase	Number of Patients	Design	OS	PFS	ORR
ILUSTRO cohort 2 [37]	NCT03505320	II	19	zolbetuximab + mFOLFOX6	-	13.7 mo 12 mo PFS 58%	63.2%
MONO [25]	NCT01197885	IIa	54	zolbetuximab monotherapy, multiple doses	-	-	23% clinical benefit rate
FAST [24]	NCT01630083	Iib	246	EOX vs. EOX + zolbetuximab	8.3 mo vs. 13	5.3 mo vs. 7.5	25% vs. 39%
SPOTLIGHT [38]	NCT03504397	III	566	Double-blind randomized mFOLFOX6 vs. mFOLFOX6 + zolbetuximab	15.5 mo vs. 18.2	8.7 mo vs. 10.6	62.1% vs. 60.7%

Legend: DCR: disease control rate; EOX: epirubicin, oxaliplatin, capecitabine; mo: months; mFOLFOX6: modified 5-fluorouracil, oxaliplatin, leucovorin; ORR: overall response rate; OS: overall survival; PFS: progression-free survival.

## Data Availability

Data sharing is not applicable.

## References

[1] Cancer Genome Atlas Research Network (2014). Comprehensive molecular characterization of gastric adenocarcinoma. Nature.

[2] Ajani J.A., D’Amico T.A., Bentrem D.J., Chao J., Cooke D., Corvera C., Das P., Enzinger P.C., Enzler T., Fanta P. (2022). Gastric Cancer, Version 2.2022, NCCN Clinical Practice Guidelines in Oncology. J. Natl. Compr. Cancer Netw..

[3] Janjigian Y.Y., Shitara K., Moehler M., Garrido M., Salman P., Shen L., Wyrwicz L., Yamaguchi K., Skoczylas T., Campos Bragagnoli A. (2021). First-line nivolumab plus chemotherapy versus chemotherapy alone for advanced gastric, gastro-oesophageal junction, and oesophageal adenocarcinoma (CheckMate 649): A randomised, open-label, phase 3 trial. Lancet.

[4] Janjigian Y.Y., Kawazoe A., Yanez P., Li N., Lonardi S., Kolesnik O., Barajas O., Bai Y., Shen L., Tang Y. (2021). The KEYNOTE-811 trial of dual PD-1 and HER2 blockade in HER2-positive gastric cancer. Nature.

[5] Shitara K., Bang Y.J., Iwasa S., Sugimoto N., Ryu M.H., Sakai D., Chung H.C., Kawakami H., Yabusaki H., Lee J. (2020). Trastuzumab Deruxtecan in Previously Treated HER2-Positive Gastric Cancer. N. Engl. J. Med..

[6] Chung H.C., Lwin Z., Gomez-Roca C., Longo F., Yanez E., Alvarez E.C., Graham D., Doherty M., Cassier P., Lopez J.S. (2021). LEAP-005: A phase II multicohort study of lenvatinib plus pembrolizumab in patients with previously treated selected solid tumors—Results from the gastric cancer cohort. J. Clin. Oncol..

[7] Salati M., Ghidini M., Paccagnella M., Reggiani Bonetti L., Bocconi A., Spallanzani A., Gelsomino F., Barbin F., Garrone O., Daniele B. (2023). Clinical Significance of Molecular Subtypes in Western Advanced Gastric Cancer: A Real-World Multicenter Experience. Int. J. Mol. Sci..

[8] Sahin U., Koslowski M., Dhaene K., Usener D., Brandenburg G., Seitz G., Huber C., Tureci O. (2008). Claudin-18 splice variant 2 is a pan-cancer target suitable for therapeutic antibody development. Clin. Cancer Res..

[9] Wang D.W., Zhang W.H., Danil G., Yang K., Hu J.K. (2022). The role and mechanism of claudins in cancer. Front. Oncol..

[10] Hashimoto I., Oshima T. (2022). Claudins and Gastric Cancer: An Overview. Cancers.

[11] Arnold A., Daum S., von Winterfeld M., Berg E., Hummel M., Rau B., Stein U., Treese C. (2020). Prognostic impact of Claudin 18.2 in gastric and esophageal adenocarcinomas. Clin. Transl. Oncol..

[12] Bardet C., Ribes S., Wu Y., Diallo M.T., Salmon B., Breiderhoff T., Houillier P., Muller D., Chaussain C. (2017). Claudin Loss-of-Function Disrupts Tight Junctions and Impairs Amelogenesis. Front. Physiol..

[13] Singh P., Toom S., Huang Y. (2017). Anti-claudin 18.2 antibody as new targeted therapy for advanced gastric cancer. J. Hematol. Oncol..

[14] Krause G., Winkler L., Mueller S.L., Haseloff R.F., Piontek J., Blasig I.E. (2008). Structure and function of claudins. Biochim. Biophys. Acta.

[15] Otani T., Furuse M. (2020). Tight Junction Structure and Function Revisited. Trends Cell Biol..

[16] Martin T.A., Jiang W.G. (2009). Loss of tight junction barrier function and its role in cancer metastasis. Biochim. Biophys. Acta.

[17] Bhat A.A., Uppada S., Achkar I.W., Hashem S., Yadav S.K., Shanmugakonar M., Al-Naemi H.A., Haris M., Uddin S. (2018). Tight Junction Proteins and Signaling Pathways in Cancer and Inflammation: A Functional Crosstalk. Front. Physiol..

[18] Swisshelm K., Macek R., Kubbies M. (2005). Role of claudins in tumorigenesis. Adv. Drug Deliv. Rev..

[19] Ding L., Lu Z., Lu Q., Chen Y.H. (2013). The claudin family of proteins in human malignancy: A clinical perspective. Cancer Manag. Res..

[20] Tureci O., Koslowski M., Helftenbein G., Castle J., Rohde C., Dhaene K., Seitz G., Sahin U. (2011). Claudin-18 gene structure, regulation, and expression is evolutionary conserved in mammals. Gene.

[21] Niimi T., Nagashima K., Ward J.M., Minoo P., Zimonjic D.B., Popescu N.C., Kimura S. (2001). claudin-18, a novel downstream target gene for the T/EBP/NKX2.1 homeodomain transcription factor, encodes lung- and stomach-specific isoforms through alternative splicing. Mol. Cell Biol..

[22] Dottermusch M., Kruger S., Behrens H.M., Halske C., Rocken C. (2019). Expression of the potential therapeutic target claudin-18.2 is frequently decreased in gastric cancer: Results from a large Caucasian cohort study. Virchows. Arch..

[23] Baek J.H., Park D.J., Kim G.Y., Cheon J., Kang B.W., Cha H.J., Kim J.G. (2019). Clinical Implications of Claudin18.2 Expression in Patients with Gastric Cancer. Anticancer Res..

[24] Sahin U., Tureci O., Manikhas G., Lordick F., Rusyn A., Vynnychenko I., Dudov A., Bazin I., Bondarenko I., Melichar B. (2021). FAST: A randomised phase II study of zolbetuximab (IMAB362) plus EOX versus EOX alone for first-line treatment of advanced CLDN18.2-positive gastric and gastro-oesophageal adenocarcinoma. Ann. Oncol..

[25] Tureci O., Sahin U., Schulze-Bergkamen H., Zvirbule Z., Lordick F., Koeberle D., Thuss-Patience P., Ettrich T., Arnold D., Bassermann F. (2019). A multicentre, phase IIa study of zolbetuximab as a single agent in patients with recurrent or refractory advanced adenocarcinoma of the stomach or lower oesophagus: The MONO study. Ann. Oncol..

[26] Pellino A., Brignola S., Riello E., Niero M., Murgioni S., Guido M., Nappo F., Businello G., Sbaraglia M., Bergamo F. (2021). Association of CLDN18 Protein Expression with Clinicopathological Features and Prognosis in Advanced Gastric and Gastroesophageal Junction Adenocarcinomas. J. Pers. Med..

[27] Resnick M.B., Konkin T., Routhier J., Sabo E., Pricolo V.E. (2005). Claudin-1 is a strong prognostic indicator in stage II colonic cancer: A tissue microarray study. Mod. Pathol..

[28] Jung H., Jun K.H., Jung J.H., Chin H.M., Park W.B. (2011). The expression of claudin-1, claudin-2, claudin-3, and claudin-4 in gastric cancer tissue. J. Surg. Res..

[29] Wang H., Yang X. (2015). The expression patterns of tight junction protein claudin-1, -3, and -4 in human gastric neoplasms and adjacent non-neoplastic tissues. Int. J. Clin. Exp. Pathol..

[30] Ren F., Zhao Q., Zhao M., Zhu S., Liu B., Bukhari I., Zhang K., Wu W., Fu Y., Yu Y. (2021). Immune infiltration profiling in gastric cancer and their clinical implications. Cancer Sci..

[31] Chen X., Zhao J., Li A., Gao P., Sun J., Song Y., Liu J., Chen P., Wang Z. (2016). Clinicopathological significance of claudin 4 expression in gastric carcinoma: A systematic review and meta-analysis. Onco Targets Ther..

[32] Kohmoto T., Masuda K., Shoda K., Takahashi R., Ujiro S., Tange S., Ichikawa D., Otsuji E., Imoto I. (2020). Claudin-6 is a single prognostic marker and functions as a tumor-promoting gene in a subgroup of intestinal type gastric cancer. Gastric Cancer.

[33] Rao X., Jiang J., Liang Z., Zhang J., Zhuang Z., Qiu H., Luo H., Weng N., Wu X. (2021). Down-Regulated CLDN10 Predicts Favorable Prognosis and Correlates with Immune Infiltration in Gastric Cancer. Front. Genet..

[34] Athauda A., Chau I. (2021). Claudin 18.2-a FAST-moving target in gastric cancer?. Ann. Oncol..

[35] Jun K.H., Kim J.H., Jung J.H., Choi H.J., Chin H.M. (2014). Expression of claudin-7 and loss of claudin-18 correlate with poor prognosis in gastric cancer. Int. J. Surg..

[36] Moentenich V., Gebauer F., Comut E., Tuchscherer A., Bruns C., Schroeder W., Buettner R., Alakus H., Loeser H., Zander T. (2020). Claudin 18.2 expression in esophageal adenocarcinoma and its potential impact on future treatment strategies. Oncol. Lett..

[37] Klempner S.J., Lee K.-W., Metges J.-P., Catenacci D.V.T., Loupakis F., Ilson D.H., Shah M.A., Shitara K., Arozullah A., Park J.W. (2021). Phase 2 study of zolbetuximab plus mFOLFOX6 in claudin 18.2-positive locally advanced or metastatic gastric or gastroesophageal junction adenocarcinoma (G/GEJ): ILUSTRO cohort 2. J. Clin. Oncol..

[38] Shitara K., Lordick F., Bang Y.-J., Enzinger P.C., Ilson D.H., Shah M.A., Cutsem E.V., Xu R.-h., Aprile G., Xu J. (2023). Zolbetuximab + mFOLFOX6 as first-line (1L) treatment for patients (pts) with claudin-18.2+ (CLDN18.2+)/HER2− locally advanced (LA) unresectable or metastatic gastric or gastroesophageal junction (mG/GEJ) adenocarcinoma: Primary results from phase 3 SPOTLIGHT study. J. Clin. Oncol..

[39] Shah M.A., Ajani J.A., Al-Batran S.-E., Bang Y.-J., Catenacci D.V.T., Enzinger P.C., Ilson D.H., Kim S.S., Lordick F., Shitara K. (2022). Zolbetuximab + CAPOX versus CAPOX in first-line treatment of claudin18.2+/HER2– advanced/metastatic gastric or gastroesophageal junction adenocarcinoma: GLOW phase 3 study. J. Clin. Oncol..

[40] Lordick F., Al-Batran S.E., Ganguli A., Morlock R., Sahin U., Tureci O. (2021). Patient-reported outcomes from the phase II FAST trial of zolbetuximab plus EOX compared to EOX alone as first-line treatment of patients with metastatic CLDN18.2+ gastroesophageal adenocarcinoma. Gastric Cancer.

[41] Couzin-Frankel J. (2013). Breakthrough of the year 2013. Cancer immunotherapy. Science.

[42] Jiang H., Shi Z., Wang P., Wang C., Yang L., Du G., Zhang H., Shi B., Jia J., Li Q. (2019). Claudin18.2-Specific Chimeric Antigen Receptor Engineered T Cells for the Treatment of Gastric Cancer. J. Natl. Cancer Inst..

[43] Zhan X., Wang B., Li Z., Li J., Wang H., Chen L., Jiang H., Wu M., Xiao J., Peng X. (2019). Phase I trial of Claudin 18.2-specific chimeric antigen receptor T cells for advanced gastric and pancreatic adenocarcinoma. J. Clin. Oncol..

[44] Qi C., Gong J., Li J., Liu D., Qin Y., Ge S., Zhang M., Peng Z., Zhou J., Cao Y. (2022). Claudin18.2-specific CAR T cells in gastrointestinal cancers: Phase 1 trial interim results. Nat. Med..

[45] QI C., Liu C., Gong J., Li J., Liu D., Ge S., Zhang M., Peng Z., Zhou J., Zhang X. (2022). Safety, tolerability, and preliminary efficacy results in patients with advanced gastric/gastroesophageal junction adenocarcinoma from a phase Ib/II study of CLDN18.2 CAR T-cell therapy (CT041). J. Clin. Oncol..

[46] Botta G.P., Becerra C.R., Jin Z., Kim D.W., Zhao D., Lenz H.-J., Ma H., Ween A., Acha P., Li Z. (2022). Multicenter phase Ib trial in the U.S. of salvage CT041 CLDN18.2-specific chimeric antigen receptor T-cell therapy for patients with advanced gastric and pancreatic adenocarcinoma. J. Clin. Oncol..

[47] Martinez M., Moon E.K. (2019). CAR T Cells for Solid Tumors: New Strategies for Finding, Infiltrating, and Surviving in the Tumor Microenvironment. Front. Immunol..

[48] Tian Y., Li Y., Shao Y., Zhang Y. (2020). Gene modification strategies for next-generation CAR T cells against solid cancers. J. Hematol. Oncol..

[49] Xu H., Li W., Lv H., Gu D., Wei X., Dai H.-j. (2022). Tandem CAR-T cells targeting CLDN18.2 and NKG2DL for treatment of gastric cancer. J. Clin. Oncol..

